# The ENmix DNA methylation analysis pipeline for Illumina BeadChip and comparisons with seven other preprocessing pipelines

**DOI:** 10.1186/s13148-021-01207-1

**Published:** 2021-12-09

**Authors:** Zongli Xu, Liang Niu, Jack A. Taylor

**Affiliations:** 1 Epidemiology Branch, National Institute of Environmental Health Sciences, NIH, MD A3-05, 111 T.W. Alexander Drive, PO Box 12233, Research Triangle Park, NC 27709 USA; 2 Division of Biostatistics and Bioinformatics, Department of Environmental and Public Health Sciences, College of Medicine, University of Cincinnati, Cincinnati, OH USA

**Keywords:** DNA methylation, Preprocessing, Pipeline, Illumina BeadChip

## Abstract

**Background:**

Illumina DNA methylation arrays are high-throughput platforms for cost-effective genome-wide profiling of individual CpGs. Experimental and technical factors introduce appreciable measurement variation, some of which can be mitigated by careful “preprocessing” of raw data.

**Methods:**

Here we describe the ENmix preprocessing pipeline and compare it to a set of seven published alternative pipelines (ChAMP, Illumina, SWAN, Funnorm, Noob, wateRmelon, and RnBeads). We use two large sets of duplicate sample measurements with 450 K and EPIC arrays, along with mixtures of isogenic methylated and unmethylated cell line DNA to compare raw data and that preprocessed via different pipelines.

**Results:**

Our evaluations show that the ENmix pipeline performs the best with significantly higher correlation and lower absolute difference between duplicate pairs, higher intraclass correlation coefficients (ICC) and smaller deviations from expected methylation level in mixture experiments. In addition to the pipeline function, ENmix software provides an integrated set of functions for reading in raw data files from mouse and human arrays, quality control, data preprocessing, visualization, detection of differentially methylated regions (DMRs), estimation of cell type proportions, and calculation of methylation age clocks. ENmix is computationally efficient, flexible and allows parallel computing. To facilitate further evaluations, we make all datasets and evaluation code publicly available.

**Conclusion:**

Careful selection of robust data preprocessing methods is critical for DNA methylation array studies. ENmix outperformed other pipelines in our evaluations to minimize experimental variation and to improve data quality and study power.

**Supplementary Information:**

The online version contains supplementary material available at 10.1186/s13148-021-01207-1.

## Introduction

Illumina Infinium Methylation BeadChip are being widely utilized to measure individual CpG methylation on an epigenome-wide scale. These arrays use probes with two different design chemistries and two fluorescence dyes to interrogate bisulfite-modified DNA. Array experiments have a number of known sources of technical variation which may account for a sizeable fraction of data variability. Careful selection of data preprocessing methods to minimize experimental variation is critical in revealing the relatively small methylation changes associated with study variables, especially the subtle variation associated with complex disease phenotypes. Although Illumina provides some basic analysis software, large scale data analysis has been facilitated by sophisticated methods and software developed by the methylation research community. We have contributed to this effort by publishing preprocessing methods for background correction [1], probe-type bias correction [2], and dye bias correction [3] and with each method provided comparisons with other available alternatives. Here we describe the combination of these methods into the ENmix preprocessing pipeline, named after our original background correction method, and describe features of the extended ENmix methylation analysis software.

It is difficult for even experienced investigators to select from among diverse methods and then implement them in their own array analysis. To help simplify analysis, combinations of different methods are often grouped into preprocessing pipelines, offering convenient means for investigators to carry out quality control and preprocessing. Although all pipelines improve ease-of-use, their relative improvements to data quality are more difficult to assess, in part because of the lack of reliable quantitative methylation metrics with which they can be compared. Here we compare the performance of the ENmix pipeline to seven other available pipelines (Illumina, SWAN, Noob, Funnorm, wateRmelon, RnBeads, and ChAMP) [1, 4–9] using large datasets that allow us to assess two fundamental features: the agreement of methylation measurements in pairs of duplicate samples, and the accuracy of measurements.

## Methods and materials

### Methylation analysis pipelines

The ENmix pipeline performs data preprocessing in a stepwise way, including ENmix background correction [1], RELIC dye bias correction [3], optional inter-array normalization, RCP probe-type bias correction [2], quality control, filtering of low-quality data points, and imputation. ENmix background correction employs a mixture of exponential and truncated normal distributions to flexibly model complex methylation signal intensities and uses a truncated normal distribution to model background noise. The RELIC dye bias correction method makes use of the internal control probes designed to monitor intensity of the red and green color channels. It first derives a relative quantitative relationship between red and green channels using the log transformed intensity values, and then use this relationship to correct for dye bias on whole array. Optional inter-array normalization is recommended for relatively homogeneous samples, i.e., where overall intensity value distributions are similar between samples. It performs quantile normalization on intensity values separately for methylated/unmethylated, red/green channel and type I/II probes. RCP probe-type bias correction employs a regression framework and uses the correlation relationship between nearby type I and II probes to calibrate the methylation beta values for type II probes. In addition to the preprocessing pipeline function, the ENmix R software provides a set of functions to facilitate large-scale epigenetic analyses including direct import of IDAT files and Illumina manifest files, quality control measures, imputation, surrogate variable analysis for batch effects using internal control probes, ICC calculation, epigenetic clocks, differential methylated region (DMR) analysis, and estimation of blood cell proportions (see Supplemental Materials and Users Guide at: https://www.bioconductor.org/packages/release/bioc/html/ENmix.html).

The Illumina pipeline, implemented in GenomeStudio Methylation Module and in the minfi software package [10], uses a background subtraction method for background correction. It first calculates the 5th percentile of the negative control intensities separately for red and green channels and then subtracts it from corresponding color channel intensities for all other probes. Probes with intensity values below than the 5th percentile are assigned a constant close to 0. For dye bias correction, the Illumina pipeline uses the first sample in a dataset as a reference to normalize red and green channel intensities for all other samples, so the ratio of intensities between color channels for internal control probes is the same across all samples.

SWAN is short for subset-quantile within array normalization [4] and is implemented in the minfi software package. It assumes that Infinium I and II probes with similar surrounding CpG density have similar measurement intensity distributions. The method first separates all probes into different groups based on CpG density, then using a random subset within each group, derives an average quantile distribution between Infinium I and II probes, and finally adjusts intensities for each probe type separately using a linear interpolation method.

The Noob background correction method utilizes a normal–exponential convolution method [11] to model methylation signal intensities and uses out-of-band probe intensities [5] (intensity reads of Infinium I probes from the color channel opposite of their design (Cy3/Cy5) to measure non-specific fluorescence) to estimate background distribution. The dye bias correction procedure in the Noob pipeline (implemented in the minfi software package) is similar to the Illumina pipeline, but uses the average intensities of color channel internal control probes across all samples as a reference.

Funnorm is short for functional normalization [6] and is a between-array normalization method. It removes unwanted variation by regressing out the first few principal components derived from the internal control probes present on the array. The normalization procedure is performed for the methylated and unmethylated, Infinium I and II probe intensities separately. In the minfi implementation of the Funnorm pipeline, Noob background correction and dye bias correction are performed before this functional normalization.

In the wateRmelon R package, the “dasen” option is the recommend normalization method [7], which first performs background adjustment by adding the offset between Type I and II probe intensities to Type I intensities, and then conducts between-array quantile normalization on methylated/unmethylated, Infinium I/II probes separately.

The RnBeads package [8] implements multiple normalization methods available in several other software packages, such as minfi and Watermelon. The default option is also the “dasen” method in wateRmelon package.

The default normalization method in ChAMP package is the BMIQ method. BMIQ is short for beta-mixture quantile dilation. It is an inter-array normalization method to adjust the beta values of Infinium II probes into a statistical distribution characteristic of Infinium I probes. BMIQ assumes that methylation values for probes on the Illumina array follows a three-state beta-mixture model.

### Evaluation data sets

Technical duplicates: As part of an existing Sister Study we assayed 128 pairs of technical duplicate blood DNA samples from women on 450 K arrays and subsequently assayed a separate set of 125 pairs of technical duplicate blood samples on EPIC arrays. Three 450 K array sample pairs and two EPIC sample pairs were excluded because one of the sample-pair members had low data quality, i.e., where low-quality methylation sites (detection *p* > 0.000001 or number of beads < 3) per sample was greater than 5 percent or the average bisulfite intensity was less than 5500. Written informed consent and blood samples were collected at recruitment and the study was approved by the institutional review boards of the National Institute of Environmental Health Sciences (NIEHS), National Institutes of Health (NIH), and the Copernicus Group. Genomic DNA was extracted from aliquots of whole blood using an automated system (Autopure LS, Gentra Systems) in the NIEHS Molecular Genetics Core Facility or using DNAQuik at BioServe Biotechnologies LTD (Beltsville,MD). One microgram of DNA from each woman was bisulfite-converted in 96-well plates using the EZ DNA Methylation Kit (Zymo Research, Orange County CA). DNA samples were randomly distributed with respect to both plates and arrays, with the additional requirement that the two duplicates of a sample were always bisulfite-converted on different plates and assayed on different arrays. All samples were tested for completion of bisulfite conversion, and converted DNA was analyzed on Illumina Human450 Methylation Arrays following the manufacturer’s protocol. 450 K arrays were assayed at the NIH Center for Inherited Disease Research (CIDR) whereas EPIC arrays were assayed at the NCI Cancer Genomics Research Laboratory.

Standardized methylation control samples: We also created and assayed a set of 39 standardized methylation control samples. Human unmethylated and fully methylated DNA was obtained from a commercial source (Zymo Research, Irving CA). Unmethylated DNA was from the HCT116 double knockout (DKO) cell line which lacks both DNA methyltransferases DNMT1 (-/-) and DNMT3b (-/-); fully methylated DNA is from this same HCT116 DKO that is enzymatically methylated at CpG sites. We mixed unmethylated and methylated samples together in different proportions to create standardized control samples with specific methylation levels: 0, 5, 10, 20, 40, 50, 60, 80, and 100% methylated. Replicates for each methylation level (n = 10, 3, 2, 3, 3, 2, 3, 3, and 10, respectively) were independently assayed on 450 K arrays at CIDR as above.

### Evaluation statistics

In the absence of experimental or measurement variation, technical duplicates should produce identical methylation values. Although correlation coefficients are often calculated between duplicate samples (e.g., across all 450 K CpG sites) to assess concordance, such coefficients can be misleading. CpG methylation in most tissues is bimodally distributed, with the majority of CpGs having methylation near 0 or 100%, and this bimodal distribution drives most of the correlation. For example, the average correlation coefficient for arrays from two unrelated individuals is greater than 0.98, making simple correlation coefficients an insensitive statistic with which to measure concordance. There are several complementary statistics that provide better measures of methylation concordance. The first is centered correlation coefficient, where the population mean methylation level for each probe is subtracted, and the correlation is calculated on the resulting residuals. The expected centered correlation coefficient for a pair of unrelated samples is 0, while the centered correlation for a pair of duplicate samples with identical methylation values is 1. The second is the absolute methylation difference between replicate measures at each CpG site, averaged across all CpG sites. Duplicate samples with identical methylation values would have an average absolute methylation difference of 0. Finally, perhaps the most commonly used statistics to evaluate data quality at the individual probe level is the intraclass correlation coefficient (ICC), which is related to the reliability of DNA methylation measures [12], and is directly correlated with study power for individual CpGs [13]. ICC values less than 0.5 are usually classified as having poor reliability, whereas those between 0.5 and 0.75, between 0.75 and 0.9, and greater than 0.9 are classified as having moderate, good, and excellent reliability, respectively. To minimize the impact of low-quality array data, we removed data points with high detection P value (greater than 10^–6^) or small number of beads (less than 3) before calculating any of the evaluation statistics. Paired samples Wilcoxon tests were used to evaluate distribution differences between ENmix and other pipelines.

## Results

### Evaluation results

**Fig. 1:**
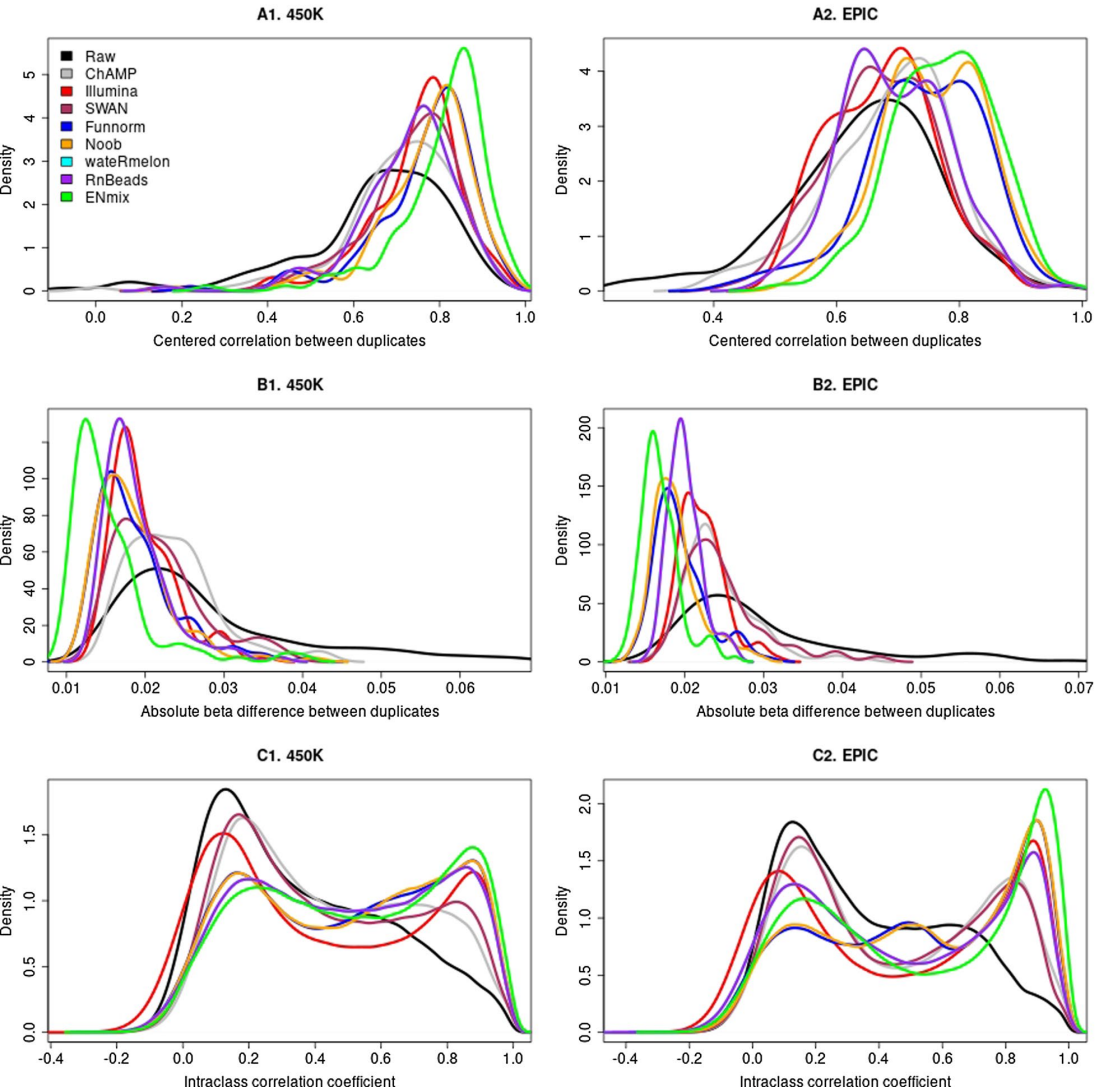
Distribution of mean-centered correlations (**A**), absolute differences (**B**), and ICC (**C**) calculated for Illumina 450 K (125 pairs) and EPIC (123 pairs) DNA methylation BeadChip in raw data and preprocessed data using various pipelines. Higher correlation, lower difference, and higher ICC indicate better performance. More detailed results are shown as violin plots in Additional file 1: Fig. S1. Paired samples Wilcoxon test P values comparing ENmix versus other pipeline distributions are shown in Additional file 1: Table S4

We applied each of the preprocessing pipelines listed in the methods to the technical duplicate datasets using each pipeline’s recommended default parameter values to evaluate how concordance between duplicates were improved (See evaluation R code in the Additional file 1). Compared to raw data from the duplicate samples run on 450 K or EPIC arrays, all pipelines provide improvements in terms of higher centered correlations (Fig. 1A, Additional file 1: Fig. S1A, and Additional file 1: Table S1) and lower average absolute differences (Fig. 1B, Additional file 1: Fig. S1B, and Additional file 1: Table S2) for duplicate pairs. The ENmix pipeline provided the highest centered correlation between duplicates (0.807 for 450 K and 0.772 for EPIC) followed by the Noob (0.769 for 450 K and 0.756 for EPIC) and Funnorm pipelines (0.765 for 450 K and 0.741 for EPIC). ENmix also provided the smallest methylation difference between duplicates (0.015 for 450 K and 0.017 for EPIC), again followed by Noob (0.018 for 450 K and 0.019 for EPIC) and Funnorm (0.019 for both 450 K and EPIC), respectively. In direct pairwise comparisons between ENmix and other pipelines, ENmix had greater centered correlation than any other pipelines for more duplicate pairs (> 87% of pairs for 450 K and > 74% for EPIC array; Additional file 1: Table S1), and smaller absolute difference than any other pipelines for more duplicate pairs (> 89% of pairs for 450 K and > 95% for EPIC; Additional file 1: Table S2; Paired samples Wilcoxon test *p* < 1 × 10^–8^ ; Additional file 1: Table S4).

We calculated ICC in the raw and preprocessed data to evaluate whether measurement reliability can be improved at probe level by each preprocessing pipeline. Figure 1C plots the distribution of ICC across all CpGs on 450 K or EPIC array using the duplicate datasets (see also Additional file 1: Fig. 1C). It again shows that, compared to raw data, all pipelines can improve ICC distributions and that the ENmix pipeline performs the best with higher overall ICCs. While only 10% of 450 K or EPIC CpGs had ICC greater than 0.75 in raw data, the ENmix pipeline improved it to 29% for 450 K and 37% for EPIC arrays, followed by the Noob and Funnorm (26% for 450 K and 34% for EPIC) pipelines. In direct comparisons between ENmix and other pipelines, the ENmix pipeline results in higher ICCs for more than 60% of CpGs on the 450 K and more than 57% CpGs on EPIC arrays (Additional file 1: Table S3; Paired samples Wilcoxon test *p* < 1 × 10^–25^, Additional file 1: Table S4).

In raw data, the methylation value distribution has four modes (Additional file 1: Fig. S2), with two modes near 0% methylation due to Infinium I and II probe-type bias, and two modes near 100% methylation due to that same bias [14]. All pipelines mitigate probe-type bias and reduce the mode differences between Infinium I and II, particularly the more substantial difference in modes near 100% methylation. However, only ENmix, SWAN and ChAMP pipelines fully adjusted these differences (Additional file 1: Fig. S2).

**Fig. 2:**
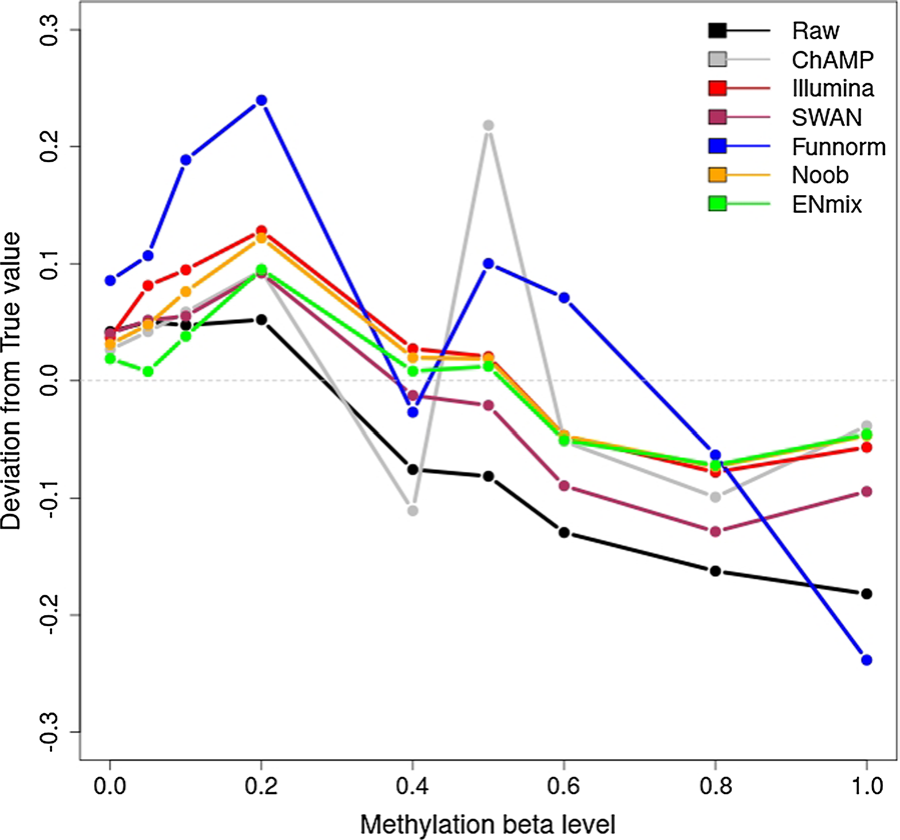
Averaged deviations of methylation distribution mode from expected methylation level for the 39 standardized methylation control samples in raw data and preprocessed data using various pipelines. Smaller deviation indicates better performance. Results for the “dasen” method (implemented in wateRmelon and RnBeads) were not shown because it is not robust in this dataset

Standardized methylation control samples created by mixing isogenic unmethylated and fully methylated DNA in various proportions provide an ordered set of nine different methylation beta value distributions, with two to ten technical replicates at each methylation level (Additional file 1: Fig. S3). Compared to raw data, four pipelines (Illumina, SWAN, Noob and ENmix) maintained proper ordering and improved the similarity among replicates samples having the same methylation level. The other four pipelines degraded the expected ordering and sometimes amplified differences among technical replicates: The default pipelines in R package RnBeads and wateRmelon produced distributions with a single narrow peak for all samples, Funnorm resulted in a disordered series and amplified differences between replicates, and ChAMP resulted in disordered and discontinuous distributions (Additional file 1: Fig. S3). In order to evaluate whether pipelines affect the accuracy of methylation measurements, we compared the methylation levels of standardized methylation control samples to the modes of the distributions obtained via each pipeline (Fig. 2). On average across all nine levels, the ENmix pipeline had the smallest absolute difference (0.038) followed by the Noob (0.053) and Illumina pipelines (0.063).

To facilitate readers’ ability to replicate our evaluation, we make all datasets and the evaluation code available at Additional file 1 and GEO (GSE174422 and GSE174390).

## Discussion

Although Illumina arrays provide a cost-effective means of measuring methylation across the genome, experimental and technical features of the arrays are known sources of measurement error and bias [5, 15]. A variety of data preprocessing methods have been developed to improve data quality, and different methods have been grouped together into convenient pipelines that facilitate data preprocessing. Comparison of data quality improvements provided by different pipelines has been problematic because robust objective standards to judge improvement have been lacking. Here we introduce the ENmix preprocessing pipeline which is based on our previously published methods for background correction, probe-type bias correction, and dye bias correction and compare it to seven other existing pipelines. As a basis for comparison we use large datasets of duplicate sample measurements from both 450 K and EPIC arrays and a dataset for standard methylation control samples. These datasets provide specific objectively true metrics that serve as gold standards for assessing data quality.

Of the eight pipelines we evaluated, ENmix and Noob performed the best, with higher methylation correlation, lower absolute difference, higher ICC and smaller deviations from true methylation levels. Both of these pipelines use model-based methods for background correction. The background correction method in the Noob pipeline assumes that intensity values are exponentially distributed and is based on the RMA method, a method originally developed for gene expression data [11]. However, the exponential distribution assumption may not fit some DNA methylation data very well [1]. Unlike gene expression profiles where the majority of genes are not expressed, the methylation profiles for Illumina DNA methylation arrays often have bimodal distributions with most CpGs having either high or low methylation. To accommodate these complex distributions, the ENmix background correction method uses a mixture of exponential and normal distributions to flexibly model array methylation signal intensity. The ENmix pipeline consistently outperform Noob in all evaluations, which demonstrated that the mixture distribution model performs better than the exponential distribution model for DNA methylation data analysis.

The Funnorm pipeline includes all the same methods used in the Noob pipeline, with an additional step of internal control surrogate variable adjustment (SVA) based on methylation intensities. Although analyses of duplicate samples showed that performance metrics for the Funnorm and Noob pipelines are quite similar, analyses of the standardized methylation control dataset showed that performance metrics for Funnorm are much poorer than Noob—indicating that the additional SVA step in Funnorm is not robust for heterogeneous samples. Robustness can be an important issue in DNA methylation data analysis because methylation profiles can be widely different for different human tissues. Numerous studies have shown that age and environmental exposures can affect a large percentage of CpGs, and thus, the overall methylation profiles of same tissue may also differ from person to person. Among all pipelines evaluated, only ENmix, Noob, Illumina, and SWAN are robust to highly heterogeneous samples.

Current Illumina arrays have Infinium I and Infinium II probes that utilize two different chemistries, and result in methylation distributions with different modes for the two probe types. Infinium I probes account for 28% of CpGs in the 450 K array and 16% of CpGs in the EPIC array. Among the pipelines evaluated here, only ENmix, SWAN and ChAMP explicitly address this issue with different methods: SWAN assumes that signal intensities for Infinium I and II probes with similar CpG density have similar distribution. ChAMP uses the BMIQ method, which assumes methylation beta values for Infinium I and II probes have similar distribution characteristics and follow a three-state beta-mixture model. As noted in the ChAMP user’s guide, the BMIQ function may fail if a sample’s methylation value distribution is not beta distributed; this failure was evident in analysis of standardized control samples (Additional file 1: Fig. S3). ENmix uses the RCP method which does not have an overall distribution assumption, but does assume that nearby (less than 50 bp distance) Infinium I and II probes located in regions with similar CpG density have similar methylation levels.

Several packages provide options to utilize identical methods for some preprocessing steps, for example, the RnBeads package provides many of the same functions that are available in wateRmelon, SWAN and minfi. We did not explicitly evaluate all available methods in each package, and we assume that the performance of the same method in different packages are similar, as we demonstrated for the “dasen” method in both the wateRmelon and RnBeads packages. Another limitation of our study is that we only evaluated the preprocessing pipelines in each package using their default settings. These default options are the developer’s recommended settings for most users. Although the default settings for the ENmix pipeline were not specifically tuned for the databases used in this study, we cannot exclude the possibility that adjusting the default setting of ENmix or the other pipelines might change their relative performance. We believe these comparisons to be unbiased, but we provide both the code and the databases in order to encourage further comparison and improvement in preprocessing methods.

The ENmix pipeline performs data preprocessing in a sequential manner, which is flexible, transparent, and easy to use. The default configurations perform dye bias correction, background correction, and probe-type bias correction. Users can choose to perform quality controls steps which include identifying and filtering low-quality and outlier samples, probes, data points, and imputing missing values. For relatively homogeneous samples we recommend including an extra step of quantile normalization using signal intensity data. To further improve data quality and reduce experimental batch effects, we also recommend adjusting plate and nonnegative internal control surrogate variables when performing association statistical analysis. In addition to data preprocessing, ENmix software also provides many other functions to facilitate methylation-related analyses with details provided in the ENmix user’s guide.

## Conclusion

DNA methylation array experiments can introduce substantial amount of data variations. Technical duplicates and standardized control samples can be used to provide objective evaluations on DNA methylation pipelines in terms of robustness and minimizing experimental noise. Our study showed that the performance of different preprocessing pipelines can vary widely, and thus, it is critical to select appropriate analysis methods in DNA methylation studies to reveal relatively small methylation changes associated with complex traits. ENmix pipeline outperformed other methods in our evaluations and resulted in more accurate and robust estimates, higher concordance between duplicate pairs and higher ICC for individual CpGs.

## Supplementary Information

**Additional file 1.** ENmix DNA methylation analysis pipeline for Illumina BeadChip and comparisons with seven other preprocessing pipelines.

## Data Availability

All Evaluation datasets and software code are available at Additional file 1 and GEO (GSE174422 and GSE174390).

## Abbreviations

ENmix: Exponential–normal mixture model
CpG: 5′-C-phosphate-G-3′
DMR: Differentially methylated regions
RELIC: Regression on logarithm of internal control probes
RCP: Regression on correlated probes
SWAN: Subset-quantile within array normalization
Noob: Normal–exponential convolution with out-of-band intensities
Funnorm: Functional normalization
ChAMP: Chip analysis methylation pipeline
ICC: Intraclass correlation coefficients
BMIQ: Beta-mixture quantile normalization method
