# Supplementary material for: The ENmix DNA methylation analysis pipeline for Illumina BeadChip and comparisons with seven other preprocessing pipelines
Source: Clin Epigenetics. 2021 Dec 9;13:216. doi: 10.1186/s13148-021-01207-1 (PMC8662917; doi:10.1186/s13148-021-01207-1)
Supplement: Supplementary file 1 — Additional file 1. ENmix DNA methylation analysis pipeline for Illumina BeadChip and comparisons with seven other preprocessing pipelines. [file 13148_2021_1207_MOESM1_ESM.docx]

**Additional file Methods**

**ENmix package updates**

The ENmix R package was originally developed for background correction for Illumina DNA methylation array^1^. To solve other issues in large-scale methylation studies, it has been expanded to include a variety of statistical methods ^2-5^ and now becomes a comprehensive R package for DNA methylation array analysis. The following are some features of the updated software package.

Data Acquisition: ENmix can directly import data from raw experimental IDAT files creating an object with content similar to the RGChannelSetExtended object in minfi ^6^, but with a much smaller file size (about 1/3 smaller). Unlike minfi, the resulting ENmix data object directly incorporates detailed probe annotation helping to avoid errors in downstream data analysis. The ENmix import function is independent from the probe annotation R data package, which allows direct use of the correct version of Illumina manifest files for newer arrays or the MouseMethylation BeadChip.

Data Preprocessing: Illumina methylation arrays are subject to a number of sources of measurement variation including background noise, dye- and probe-type bias, and array hybridization. ENmix provides data preprocessing methods that effectively mitigate these effects including ENmix background correction^1^, RELIC dye bias correction^2^, between-array quantile normalization and RCP probe type bias adjustment ^7^.

Quality Control: Utilizing data from internal-control and out-of-band probes, ENmix provides functions that facilitate quality control both before and after data preprocessing. Low quality samples and probes, and outlier samples can be easily identified and excluded before data preprocessing. After data preprocessing, additional low quality and outlier data points can be identified and removed. ENmix provides an option for robust imputation of missing values, so that principal component and other statistical methods that do not allow for missing data can still be used. ENmix provides an empirical method to identify “gap probes” where SNPs in probe regions affect hybridization efficiency and result in multi-modal methylation distributions.

Differential Methylated Region (DMR) Analysis: Nearby CpGs often have similar methylation, so that multi-probe analysis offers potential increases in study power. ENmix provides two complementary functions to identify DMR that are easy to use: one is a modified comb-p method, which can identify groups of CpGs with modest individual p values; another is ipDMR which utilizes interval P values and has a much lower false positive rate than comb-p ^4^.

Visualization of results: Many functions in ENmix provide visualization options to help in data analysis. These options are designed for large data sets, computationally efficient, and produce fast high-quality graphics. For example, plots of internal control-probe can help investigators understand experiment quality; fast and accurate frequency polygon plots allow inspection of data distributions; quality control plots help identify low quality samples or probes; principal component plots provide information on data variance structure; Manhattan, Q-Q and plots of genomic regions facilitate DMR and EWAS analysis.

Other functions: ENmix incorporates many commonly used methods to facilitate related DNA methylation data analyses including: estimation of 5-methylcytosine(5mC) and 5-hydroxymethylcytosine (5hmC) using sequencing or array data from paired bisulfite and oxidative bisulfite treated DNA experiments ^3^; surrogate variable analysis to adjust for batch effects using internal-control probes; use of duplicate samples to calculate intra-class correlation (ICC) to evaluate reliability of individual CpGs; estimation of human blood cell types; and calculation of methylation age using various epigenetic clocks.

**Additional file Table 1.** Comparisons of centered correlations of DNA methylation values between duplicate pairs across different preprocessing pipelines for Illumina Beadchip 450K (N=125) and EPIC (N=123)

|  | Mean centered correlation | |  | # Pairs (%) where ENmix has higher correlation | |
| --- | --- | --- | --- | --- | --- |
|  | 450K | EPIC |  | 450K | EPIC |
| Raw | 0.649 | 0.645 |  | 119 (95%) | 119(97%) |
| ChAMP | 0.709 | 0.690 |  | 121 (97%) | 113(92%) |
| Illumina | 0.743 | 0.677 |  | 113 (90%) | 119(97%) |
| SWAN | 0.731 | 0.679 |  | 115 (92%) | 119(97%) |
| Funnorm | 0.765 | 0.741 |  | 114 (91%) | 105(85%) |
| Noob | 0.769 | 0.756 |  | 112 (90%) | 97(79%) |
| WateRmelon | 0.726 | 0.697 |  | 120 (96%) | 122(99%) |
| RnBeads | 0.726 | 0.697 |  | 120 (96%) | 122(99%) |
| ENmix | 0.807 | 0.772 |  |  |  |

**Additional file Table 2.** Comparisons of absolute differences of DNA methylation values between duplicate pairs across different preprocessing pipelines for Illumina Beadchip 450K (N=125) and EPIC (N=123)

|  | Mean absolute difference | |  | # Pairs (%) where ENmix has smaller difference | |
| --- | --- | --- | --- | --- | --- |
|  | 450K | EPIC |  | 450K | EPIC |
| Raw | 0.028 | 0.030 |  | 119 (95%) | 122 (99%) |
| ChAMP | 0.023 | 0.024 |  | 122 (98%) | 122 (99%) |
| Illumina | 0.020 | 0.022 |  | 114 (91%) | 122 (99%) |
| SWAN | 0.022 | 0.025 |  | 118 (94%) | 122 (99%) |
| Funnorm | 0.019 | 0.019 |  | 120 (96%) | 120 (98%) |
| Noob | 0.018 | 0.019 |  | 116 (93%) | 118 (96%) |
| WateRmelon | 0.019 | 0.020 |  | 119 (95%) | 122 (99%) |
| RnBeads | 0.019 | 0.020 |  | 119 (95%) | 122 (99%) |
| ENmix | 0.015 | 0.017 |  |  |  |

**Additional file Table 3.** Comparisons of intraclass correlation coefficients (ICC) between duplicate pairs across different preprocessing pipelines for Illumina Beadchip 450K (N=125) and EPIC (N=123)

|  | % of CpGs with ICC > 0.75 | |  | % of CpGs where ENmix has higher ICC | |
| --- | --- | --- | --- | --- | --- |
|  | 450K | EPIC |  | 450K | EPIC |
| Raw | 10% | 10% |  | 82% | 83% |
| ChAMP | 17% | 25% |  | 74% | 77% |
| Illumina | 23% | 30% |  | 75% | 75% |
| SWAN | 19% | 23% |  | 77% | 81% |
| Funnorm | 26% | 34% |  | 61% | 58% |
| Noob | 26% | 34% |  | 60% | 57% |
| WateRmelon | 25% | 28% |  | 63% | 72% |
| RnBeads | 25% | 28% |  | 63% | 72% |
| ENmix | 29% | 37% |  |  |  |

**Additional file Table 4.** Paired samples Wilcoxon test P values from comparisons between ENmix and other pipelines for mean-centered correlation, absolute difference, and ICC based on distributions shown in Additional file Figure 1.

|  | 450K Array | | |  | EPIC Array | | |
| --- | --- | --- | --- | --- | --- | --- | --- |
|  | Correlation | Difference | ICC |  | Correlation | Difference | ICC |
| Raw | 2.90×10^-21^ | 4.97×10^-21^ | <1.00×10^-25^ |  | 1.44×10-^20^ | 6.56×10^-22^ | <1.00×10^-25^ |
| ChAMP | 6.14×10^-21^ | 4.53×10^-21^ | <1.00×10^-25^ |  | 1.98×10^-19^ | 7.06×10^-22^ | <1.00×10^-25^ |
| Illumina | 5.08×10^-17^ | 5.76×10^-17^ | <1.00×10^-25^ |  | 1.16×10^-20^ | 7.98×10^-22^ | <1.00×10^-25^ |
| SWAN | 9.09×10^-20^ | 3.84×10^-19^ | <1.00×10^-25^ |  | 8.34×10^-21^ | 6.89×10^-22^ | <1.00×10^-25^ |
| Funnorm | 1.01×10^-16^ | 4.92×10^-20^ | <1.00×10^-25^ |  | 1.53×10^-15^ | 1.21×10^-21^ | <1.00×10^-25^ |
| Noob | 4.53×10^-16^ | 1.35×10^-18^ | <1.00×10^-25^ |  | 1.87×10^-09^ | 5.42×10^-21^ | <1.00×10^-25^ |
| wateRmelon | 4.64×10^-21^ | 3.51×10^-19^ | <1.00×10^-25^ |  | 7.24×10^-22^ | 7.06×10^-22^ | <1.00×10^-25^ |
| RnBeads | 4.64×10^-21^ | 3.51×10^-19^ | <1.00×10^-25^ |  | 7.24×10^-22^ | 7.06×10^-22^ | <1.00×10^-25^ |

**Additional file Figure 1.** Violin plots showing distributions of mean-centered correlations (A), absolute differences (B) and ICC (C) calculated for Illumina 450K (125 pairs) and EPIC (123 pairs) DNA methylation BeadChip in raw data and after preprocessed using various pipelines. Each violin plot shows median (open circle), interquartile range (black bar) and distribution probability density. Paired samples Wilcoxon test P values for ENmix vs other pipeline distributions are shown in Additional file Table 4.


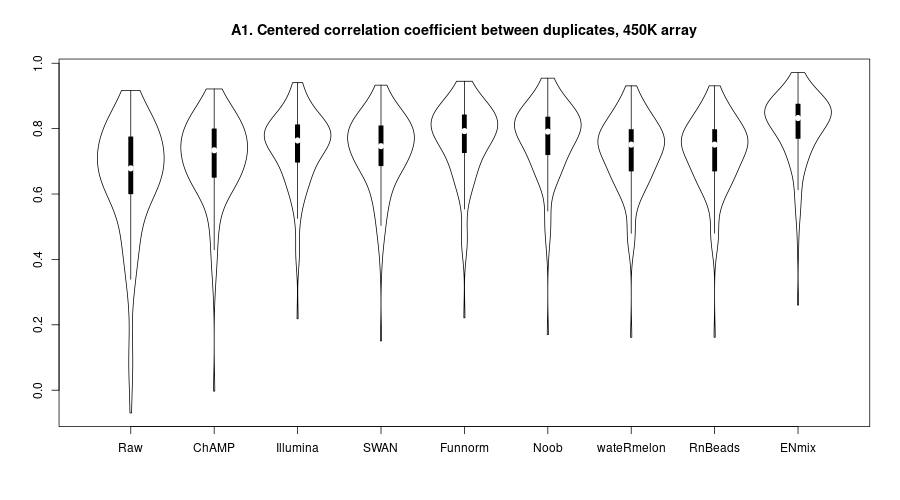


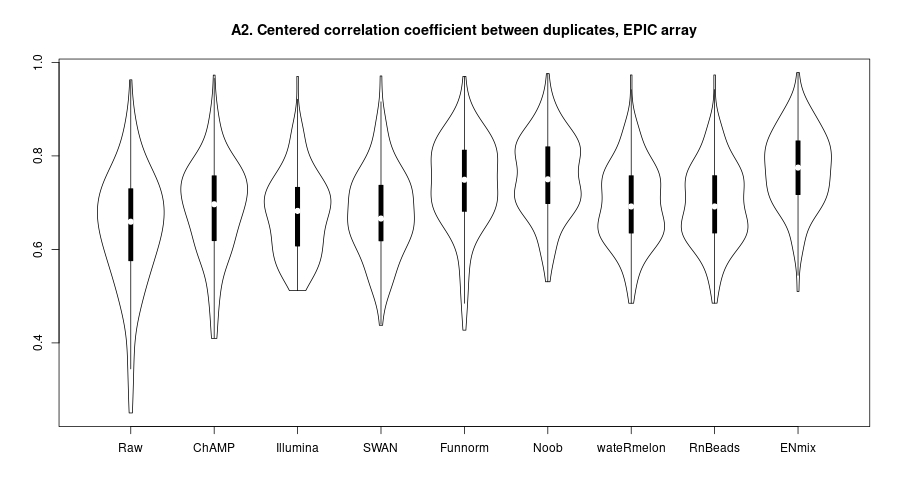


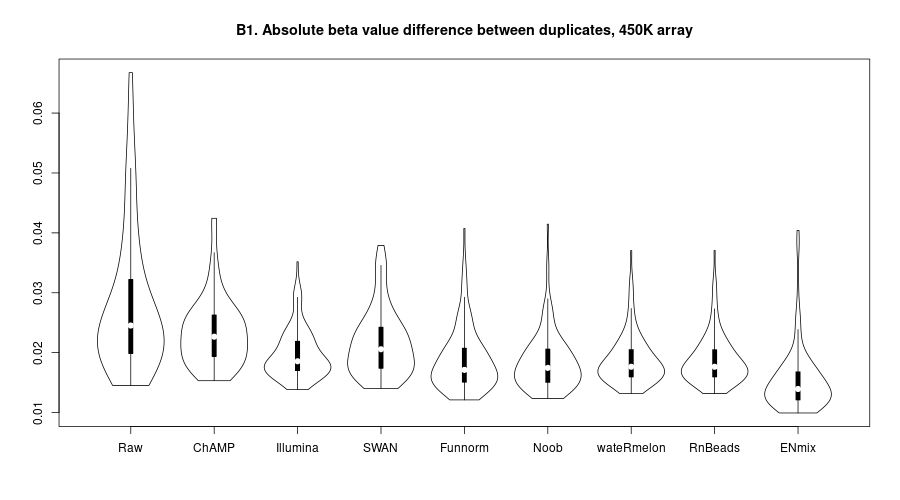


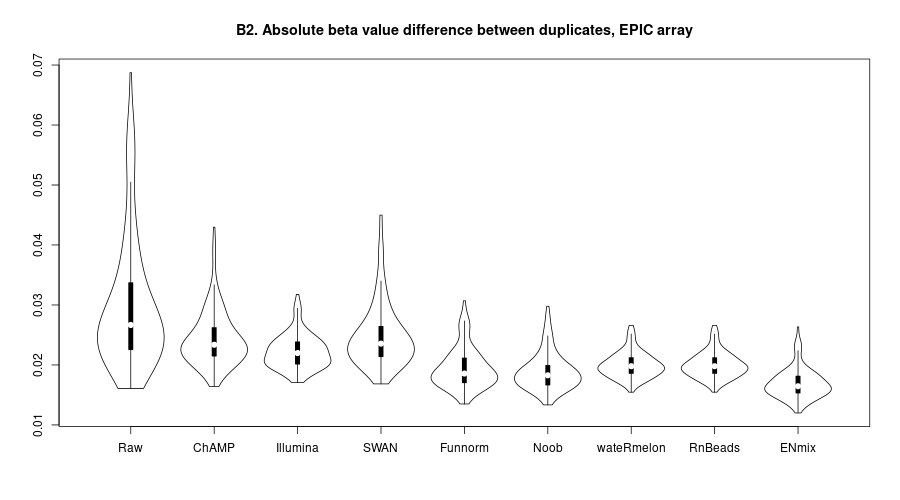


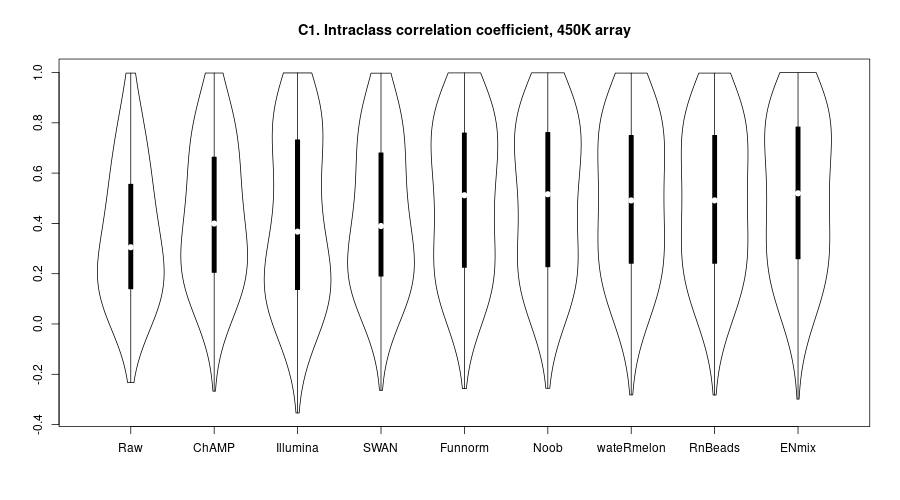

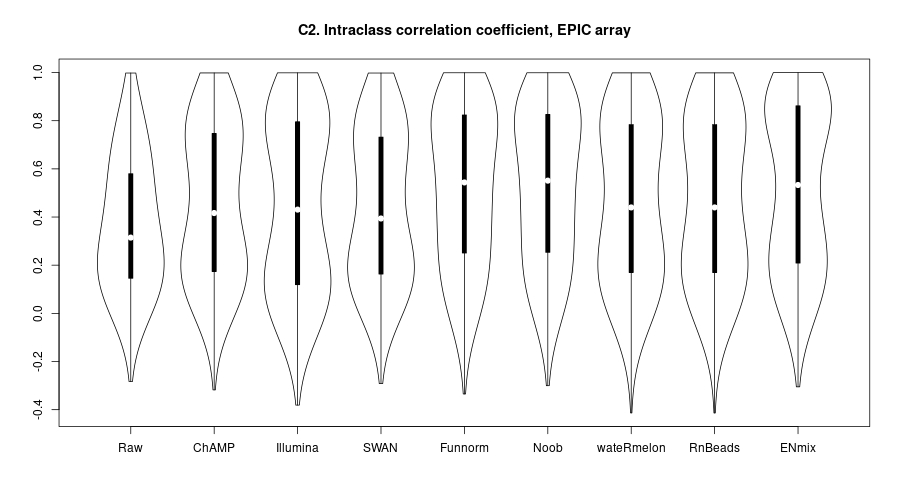


**Additional file Figure 2**. Methylation beta value distribution for 256 duplicate samples in raw data and after preprocessing by various pipelines.


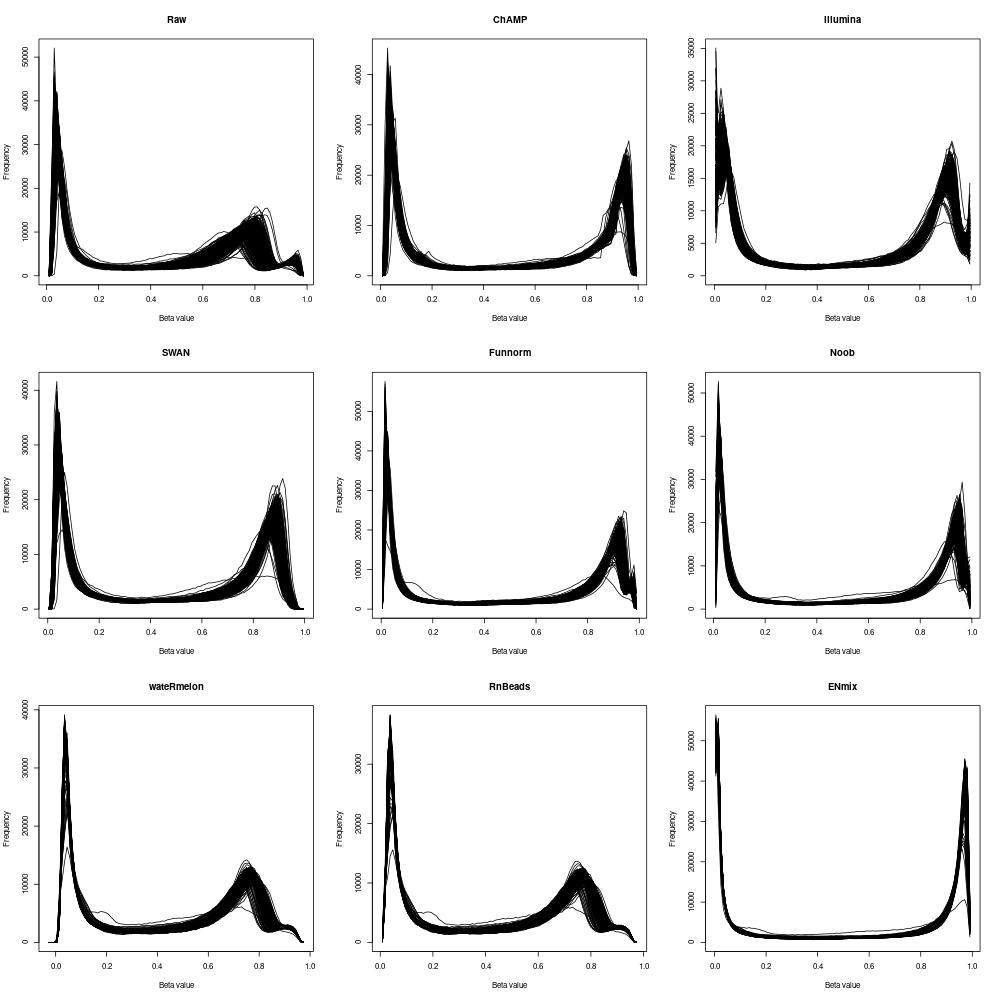


**Additional file Figure 3.** Methylation beta value distribution for 39 standardized methylation control samples in raw data and after preprocessing by various pipelines. For better comparison among the 3 top performed pipelines (Enmix, Noob and Illumina) in this dataset, we did not elect probe-type bias correlation for ENmix pipeline. Adding RCP probe type bias correction step can further improve the absolute difference for 6 out of 9 control groups.

**
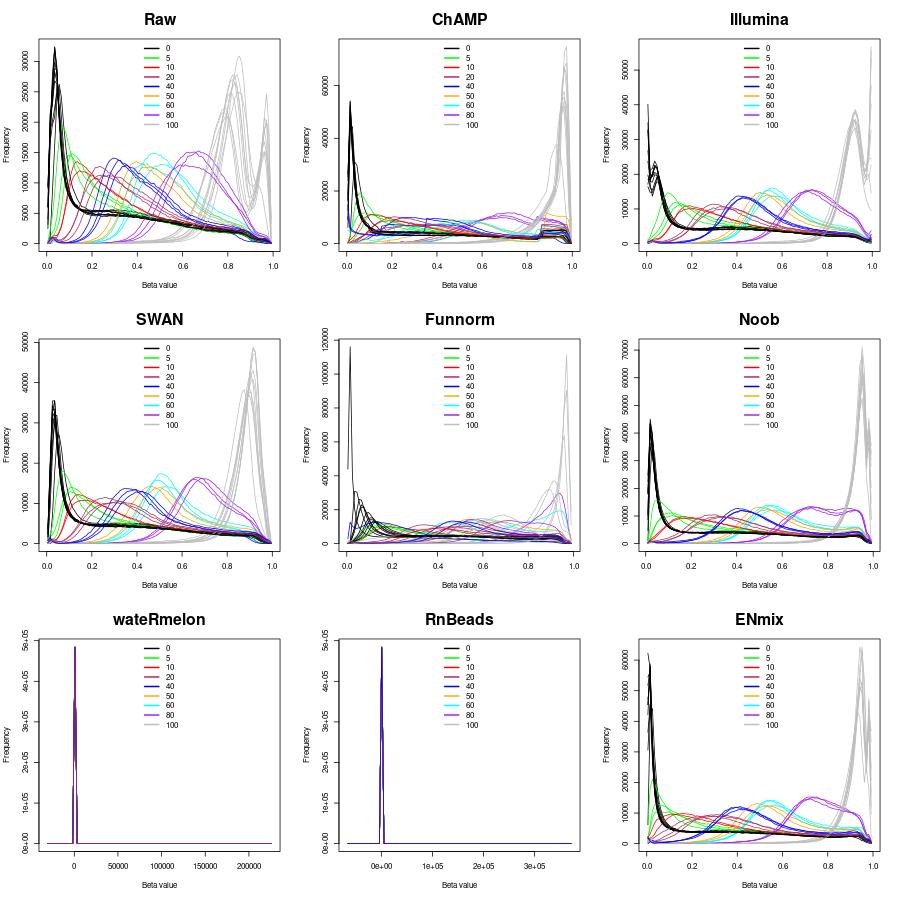
**

**Additional file code 1**. Methylation preprocessing R code used for evaluation of the eight pipelines. Duplicates datasets and annotation files can be downloaded from GEO (GSE174422).

library(ENmix)

library(minfi)

library(ChAMP)

library(wateRmelon)

library(RnBeads)

library(GEOquery)

#retrieving of annotation files from GEO, users can also manually download the data from GEO website

GSE174422 <- getGEO('GSE174422',GSEMatrix=TRUE)

dupid=pData(phenoData(GSE174422[[1]]))[,c("source_name_ch1","characteristics_ch1")]

names(dupid)=c("id1","id2")

dupid$id2=sub("duplicate_id: ","",as.vector(dupid$id2))

idx=data.frame(x1=rownames(dupid),x2=as.vector(dupid$id1))

idx$x3=paste0(idx$x1,"_",idx$x2)

rownames(idx)=as.vector(idx$x2)

dupid$idx=apply(dupid,1,function(x){paste(sort(x),collapse=",")})

dupid=dupid[!duplicated(dupid$idx),]

dupid=dupid[,c("id1","id2")]

dupid$id1=as.vector(idx[as.vector(dupid$id1),]$x3)

dupid$id2=as.vector(idx[as.vector(dupid$id2),]$x3)

#retrieving idat files from GEO, users can also manually download the data from GEO website

getGEOSuppFiles("GSE174422",filter_regex="RAW")

system("tar xf ./GSE174422/GSE174422_RAW.tar -C ./GSE174422")

system("gunzip ./GSE174422/*.gz")

baseDir="./GSE174422"

#extract quality control information

rgSet=readidat (path = baseDir, recursive = TRUE, verbose = FALSE)

qcdat=QCinfo(rgSet)

#raw data

mdat<-getmeth(rgSet)

beta_raw=getB(mdat)

#ENmix

rgSet=readidat (path = baseDir, recursive = TRUE, verbose = FALSE)

beta_enmix=mpreprocess(rgSet, fqcfilter=FALSE, nCores=20)

#illumina

rgSet <- read.metharray.exp(base = baseDir,targets = NULL, extended = TRUE, recursive=TRUE)

mdat=preprocessIllumina(rgSet)

beta_illumina=getBeta(mdat)

#SWAN

rgSet <- read.metharray.exp(base = baseDir,targets = NULL, extended = TRUE, recursive=TRUE)

mdat <- preprocessSWAN(rgSet)

beta_swan=getBeta(mdat)

#Noob

rgSet <- read.metharray.exp(base = baseDir,targets = NULL, extended = TRUE, recursive=TRUE)

mdat <- preprocessNoob(rgSet)

beta_noob=getBeta(mdat)

#Funnorm

rgSet <- read.metharray.exp(base = baseDir,targets = NULL, extended = TRUE, recursive=TRUE)

mdat <- preprocessFunnorm(rgSet)

beta_funnorm=getBeta(mdat)

#ChAMP

myLoad <- champ.load(baseDir,filterDetP=FALSE,filterBeads=FALSE,filterNoCG=TRUE, filterSNPs=FALSE,filterMultiHit=FALSE,filterXY=FALSE,autoimpute=FALSE)

beta_champ=champ.norm(beta=myLoad$beta, cores=5)

#wateRmelon

mlumi <- readEPIC(baseDir)

beta <- dasen(mlumi)

beta_watermelon=assayData(beta)$betas

#RnBeads

idat.dir =baseDir

system("rm -rvf ./rnbout")

report.dir="./rnbout"

tmp=strsplit(as.vector(idx$x2),"_")

tmp=do.call(rbind, tmp)

sample.annotation=data.frame(Sample_Name=idx$x3,Sentrix_ID=paste0(as.vector(idx$x1),"_",tmp[,1]),Sentrix_Position=tmp[,2])

write.table(sample.annotation,"sample.annotation.csv",row.names=FALSE,sep=",")

sample.annotation="sample.annotation.csv"

data.source <- c(idat.dir, sample.annotation)

result <- rnb.run.import(data.source=data.source, data.type="idat.dir", dir.reports=report.dir)

rnb.set <- result$rnb.set

rnb.set.norm<-rnb.execute.normalization(rnb.set)

beta= meth(rnb.set.norm)

rownames(beta)=rownames(annotation(rnb.set.norm))

colnames(beta)=pheno(rnb.set.norm)$Basename

beta_rnbeads=beta

#Evaluation,ICC and centered correlation

coriccr_raw<-dupicc(dat=beta_raw,dupid=dupid,center=TRUE,qcflag=TRUE,qc=qcdat,nCores=5)

coricc_enmix<-dupicc(dat=beta_enmix,dupid=dupid,center=TRUE,qcflag=TRUE,qc=qcdat,nCores=5)

coricc_illumina<-dupicc(dat=beta_illumina,dupid=dupid,center=TRUE,,qcflag=TRUE,qc=qcdat,nCores=5)

coricc_swan<-dupicc(dat=beta_swan,dupid=dupid,center=TRUE,qcflag=TRUE,qc=qcdat,nCores=5)

coricc_noob<-dupicc(dat=beta_noob,dupid=dupid,center=TRUE,qcflag=TRUE,qc=qcdat,nCores=5)

coricc_funnorm<-dupicc(dat=beta_funnorm,dupid=dupid,center=TRUE,qcflag=TRUE,qc=qcdat,nCores=5)

coricc_champ<-dupicc(dat=beta_champ,dupid=dupid,center=TRUE,qcflag=TRUE,qc=qcdat,nCores=5)

coricc_watermelon<-dupicc(dat=beta_watermelon,dupid=dupid,center=TRUE,qcflag=TRUE,qc=qcdat,nCores=5)

coricc_rnbeads<-dupicc(dat=beta_rnbeads,dupid=dupid,center=TRUE,qcflag=TRUE,qc=qcdat,nCores=5)

**Additional file code 2**. Methylation preprocessing R code used for evaluation of the eight pipelines. Standard control datasets and annotation files can be downloaded from GEO (GSE174390).

library(ENmix)

library(minfi)

library(ChAMP)

library(wateRmelon)

library(RnBeads)

library(GEOquery)

#retrieving annotation files from GEO, users can also manually download the data from GEO website

GSE174390 <- getGEO('GSE174390',GSEMatrix=TRUE)

pp=pData(phenoData(GSE174390[[1]]))[,c("source_name_ch1","characteristics_ch1")]

names(pp)=c("Sample_name","stdlevel")

pp$stdlevel=sub("methylation level \\(%\\): ","",as.vector(pp$stdlevel))

pp$Sample_name=paste0(rownames(pp),"_",as.vector(pp$Sample_name))

rownames(pp)=pp$Sample_name

#retrieve idat files from GEO, users can also manually download the data from GEO website

getGEOSuppFiles("GSE174390",filter_regex="RAW")

system("tar xf ./GSE174390/GSE174390_RAW.tar -C ./GSE174390")

system("gunzip ./GSE174390/*.gz")

baseDir="./GSE174390"

#extract quality control information

rgSet=readidat (path = baseDir, recursive = TRUE, verbose = FALSE)

qcdat=QCinfo(rgSet)

#raw data

mdat<-getmeth(rgSet)

beta_raw=getB(mdat)

#ENmix

rgSet=readidat (path = baseDir, recursive = TRUE, verbose = FALSE)

mdat <- preprocessENmix(rgSet,nCores = 20)

beta_enmix=getB(mdat)

#illumina

rgSet <- read.metharray.exp(base = baseDir,targets = NULL, extended = TRUE, recursive=TRUE)

mdat=preprocessIllumina(rgSet)

beta_illumina=getBeta(mdat)

#SWAN

rgSet <- read.metharray.exp(base = baseDir,targets = NULL, extended = TRUE, recursive=TRUE)

mdat <- preprocessSWAN(rgSet)

beta_swan=getBeta(mdat)

#Noob

rgSet <- read.metharray.exp(base = baseDir,targets = NULL, extended = TRUE, recursive=TRUE)

mdat <- preprocessNoob(rgSet)

beta_noob=getBeta(mdat)

#Funnorm

rgSet <- read.metharray.exp(base = baseDir,targets = NULL, extended = TRUE, recursive=TRUE)

mdat <- preprocessFunnorm(rgSet)

beta_funnorm=getBeta(mdat)

#ChAMP

myLoad <- champ.load(baseDir,filterDetP=FALSE,filterBeads=FALSE,filterNoCG=TRUE, filterSNPs=FALSE,filterMultiHit=FALSE,filterXY=FALSE,autoimpute=FALSE)

beta_champ=champ.norm(beta=myLoad$beta, cores=5)

#wateRmelon

mlumi <- readEPIC(baseDir)

beta <- dasen(mlumi)

beta_watermelon=assayData(beta)$betas

#RnBeads

idat.dir =baseDir

system("rm -rvf ./rnbout")

report.dir="./rnbout"

tmp=strsplit(pp$Sample_name,"_")

tmp=do.call(rbind, tmp)

sample.annotation=data.frame(Sample_Name=pp$Sample_name,Sentrix_ID=paste0(tmp[,1],"_",tmp[,2]),Sentrix_Position=tmp[,3])

write.table(sample.annotation,"sample.annotation.csv",row.names=FALSE,sep=",")

sample.annotation="sample.annotation.csv"

data.source <- c(idat.dir, sample.annotation)

result <- rnb.run.import(data.source=data.source, data.type="idat.dir", dir.reports=report.dir)

rnb.set <- result$rnb.set

rnb.set.norm<-rnb.execute.normalization(rnb.set)

beta= meth(rnb.set.norm)

rownames(beta)=rownames(annotation(rnb.set.norm))

colnames(beta)=pheno(rnb.set.norm)$Basename

beta_rnbeads=beta

#Evaluations

#Standadized sample id

pp$col="black"

pp$col[pp$stdlevel==5]="green"

pp$col[pp$stdlevel==10]="red"

pp$col[pp$stdlevel==20]="maroon"

pp$col[pp$stdlevel==40]="blue"

pp$col[pp$stdlevel==50]="orange"

pp$col[pp$stdlevel==60]="cyan"

pp$col[pp$stdlevel==80]="purple"

pp$col[pp$stdlevel==100]="grey"

colcode=c("black","green","red","maroon","blue","orange","cyan","purple","grey")

mode <- function(x) {

x=x[!is.na(x)]

lim.inf=min(x); lim.sup=max(x)

s<-density(x,from=lim.inf,to=lim.sup)

n<-length(s$y)

v1<-s$y[1:(n-2)];

v2<-s$y[2:(n-1)];

v3<-s$y[3:n]

ix<-1+which((v1<v2)&(v2>v3))

md <- s$x[which(s$y==max(s$y))]

return(md)

}

allmode=pp[,c("Sample_name","stdlevel")]

method=c("raw","champ","illumina","swan","funnorm","noob","watermelon","rnbeads","enmix")

jpeg("dist_std.jpg",height=900,width=900)

par(mfrow=c(3,3))

for(md in method){

beta=get(paste0("beta_",md))

beta=qcfilter(beta,qcscore=qcdat,rmoutlier=FALSE)

pp=pp[colnames(beta),]

multifreqpoly(beta,col=pp$col,main=md,xlab="Beta value",legend="",cex.main=2);

legend("top", c("0","5","10","20","40","50","60","80","100"), col=colcode,lty=1,lwd=2,horiz=FALSE,bty="n",cex=1)

m=apply(beta,2,mode)

m=data.frame(Sample_name=names(m),mode=m)

allmode=merge(allmode,m,by="Sample_name")

names(allmode)[ncol(allmode)]=md

}

dev.off()

**References**

1. Xu Z, Niu L, Li L, Taylor JA. ENmix: a novel background correction method for Illumina HumanMethylation450 BeadChip. Nucleic Acids Res 2016; 44:e20.

2. Xu Z, Langie SA, De Boever P, Taylor JA, Niu L. RELIC: a novel dye-bias correction method for Illumina Methylation BeadChip. BMC Genomics 2017; 18:4.

3. Xu Z, Taylor JA, Leung YK, Ho SM, Niu L. oxBS-MLE: an efficient method to estimate 5-methylcytosine and 5-hydroxymethylcytosine in paired bisulfite and oxidative bisulfite treated DNA. Bioinformatics 2016; 32:3667-9.

4. Xu Z, Xie C, Taylor JA, Niu L. ipDMR: Identification of differentially methylated regions with interval p-values. Bioinformatics 2020.

5. Xu ZL, Sandler DP, Taylor JA. Blood DNA Methylation and Breast Cancer: A Prospective Case-Cohort Analysis in the Sister Study. Jnci-J Natl Cancer I 2020; 112:87-94.

6. Fortin JP, Triche TJ, Jr., Hansen KD. Preprocessing, normalization and integration of the Illumina HumanMethylationEPIC array with minfi. Bioinformatics 2017; 33:558-60.

7. Niu L, Xu Z, Taylor JA. RCP: a novel probe design bias correction method for Illumina Methylation BeadChip. Bioinformatics 2016; 32:2659-63.
